# Supplementary material for: Multivalent coiled-coil interactions enable full-scale centrosome assembly and strength
Source: J Cell Biol. 2024 Mar 8;223(4):e202306142. doi: 10.1083/jcb.202306142 (PMC10921949; doi:10.1083/jcb.202306142)
Supplement: Table S1 — lists constructs for protein expression. [file JCB_202306142_TableS1.docx]

**TABLE S1. Constructs for protein expression.**

| **protein** | **sequence** | **plasmid name** | **parent plasmid** | **N-term tag** | **C-term tag** |
| --- | --- | --- | --- | --- | --- |
| SPD-5 | Full-length | JWV2 | pOCC28 | MBP-PreScission | PreScission-6xHis |
| SPD-5::GFP | Full-length | JWV1 | pOCC27 | MBP-PreScission | **eGFP**-PreScission-6xHis |
| SPD-5::RFP | Full-length | JWV3 | pOCC25 | MBP-PreScission | tagRFP-PreScission-6xHis |
| SPD-5::GFP | (R592K) | JWV13 | pOCC27 | MBP-PreScission | **eGFP**-PreScission-6xHis |
| SPD-5 | a.a. 541-677 | JWB127 | 6xHis_1B | 6xHis-TEV |  |
| SPD-5 | a.a. 541-677 (R592K) | JWB153 | 6xHis_1B | 6xHis-TEV |  |
| SPD-5 (F22) | aa 281-386 | JWV22 | pOCC27 | MBP-PreScission | **eGFP**-PreScission-6xHis |
| SPD-5 (F23) | aa 386-566 | JWV23 | pOCC27 | MBP-PreScission | **eGFP**-PreScission-6xHis |
| SPD-5 (F24) | aa 566-1198 | JWV24 | pOCC27 | MBP-PreScission | **eGFP**-PreScission-6xHis |
| SPD-5 (F25) | aa 730-990 | JWV25 | pOCC27 | MBP-PreScission | **eGFP**-PreScission-6xHis |
| SPD-5 (F26) | aa 730-1198 | JWV26 | pOCC27 | MBP-PreScission | **eGFP**-PreScission-6xHis |
| SPD-5 (F27) | aa 990-1198 | JWV27 | pOCC27 | MBP-PreScission | **eGFP**-PreScission-6xHis |
| SPD-5 (ΔCC-LONG) | a.a. 734-918 deleted | JWV118 | pOCC27 | MBP-PreScission | **eGFP**-PreScission-6xHis |
| SPD-5 (F20) | aa 1-386 |  | pOCC28 | MBP-PreScission | PreScission-6xHis |
| SPD-5 (F21) | aa 1-566 |  | pOCC28 | MBP-PreScission | PreScission-6xHis |
| SPD-5 (F22) | aa 281-386 |  | pOCC28 | MBP-PreScission | PreScission-6xHis |
| SPD-5 (F23) | aa 386-566 |  | pOCC28 | MBP-PreScission | PreScission-6xHis |
| SPD-5 (F24) | aa 566-1198 |  | pOCC28 | MBP-PreScission | PreScission-6xHis |
| SPD-5 (F25) | aa 730-990 |  | pOCC28 | MBP-PreScission | PreScission-6xHis |
| SPD-5 (F26) | aa 730-1198 |  | pOCC28 | MBP-PreScission | PreScission-6xHis |
| SPD-5 (F27) | aa 990-1198 |  | pOCC28 | MBP-PreScission | PreScission-6xHis |
| PLK-1(CA) | T194D | JWV11 | pOCC7 | 6xHis-PreScission |  |
| PLK-1(KD) | K67M | JWV12 | pOCC7 | 6xHis-PreScission |  |
