# Supplementary material for: Multivalent coiled-coil interactions enable full-scale centrosome assembly and strength
Source: J Cell Biol. 2024 Mar 8;223(4):e202306142. doi: 10.1083/jcb.202306142 (PMC10921949; doi:10.1083/jcb.202306142)
Supplement: Table S2 — lists the C. elegans strains used in this study. [file JCB_202306142_TableS2.docx]

**TABLE S2. C. elegans strains.**

| Strain name | genotype | Creation method | Origin |
| --- | --- | --- | --- |
| JWW225-R592K | *utswSi14[pVV103/ pOD1021; Pspd-2::GFP::SPD-5(R592K);cb-unc-119(+)]II* | MosSCI, into EG6699 | This study |
| EU856 | *spd-5(or213) I.* | EMS | (Hamill et al., 2002) |
| JWW134-F21 | *utswSi12[pVV103/ pOD1021; Pspd-2::GFP::SPD-5(1-566);cb-unc-119(+)]II;* | MosSCI, into EG6699 | This study |
| JWW136-F24 | *utswSi13[pVV103/ pOD1021; Pspd-2::GFP::SPD-5(566-1198);cb-unc-119(+)]II;* | MosSCI, into EG6699 | This study |
| EG6699 | *ttTi5605 II; unc-119(ed3) III; oxEx1578.* |  | CGC |
| JLF359-FL | spd-5(wow36[tagrfp-t^3xmyc::spd-5]) I | CRISPR | (Magescas et al., 2019) |
| PHX5737-CC-LONG | spd-5(wow36 *syb5737[Δ734-918]) I* | CRISPR | SunyBiotech |
| PHX5763-Hairpin | spd-5(wow36 *syb5763[Δ610-640]) I* | CRISPR | This study, SunyBiotech |
