# Supplementary figures and images for: Multivalent coiled-coil interactions enable full-scale centrosome assembly and strength
Source: J Cell Biol. 2024 Mar 8;223(4):e202306142. doi: 10.1083/jcb.202306142 (PMC10921949; doi:10.1083/jcb.202306142)

**Figure 1D**

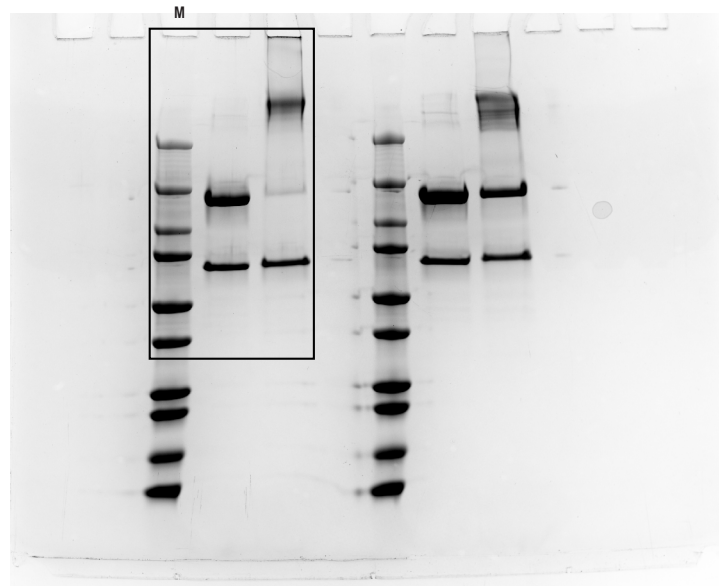

Supplement: SourceData F1 — is the source file for Fig. 1. [file JCB_202306142_SourceDataF1.pdf]

Figure 2A

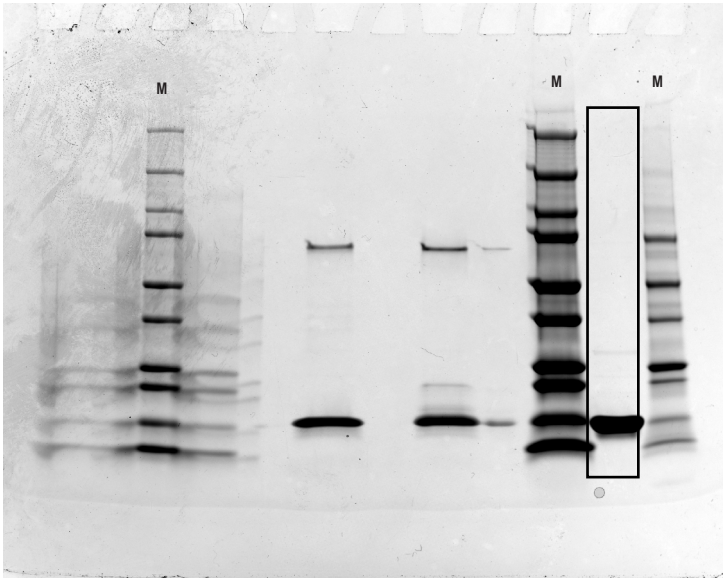

Figure 2E

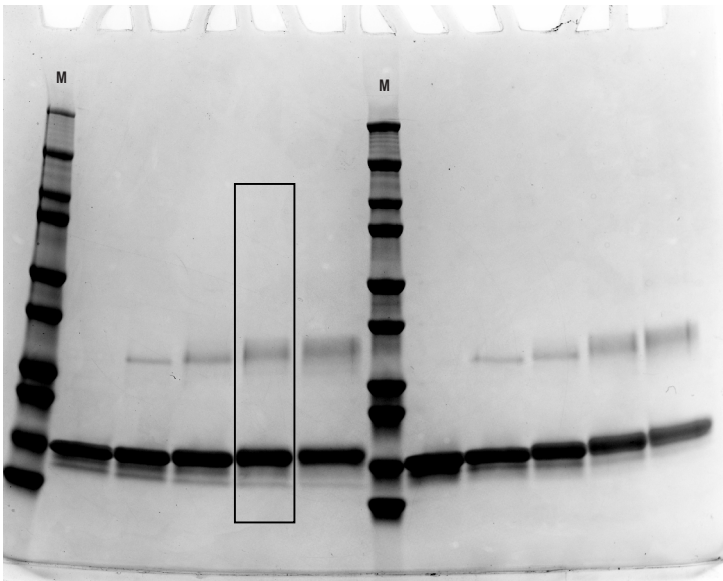

Supplement: SourceData F2 — is the source file for Fig. 2. [file JCB_202306142_SourceDataF2.pdf]

**Figure S2C**

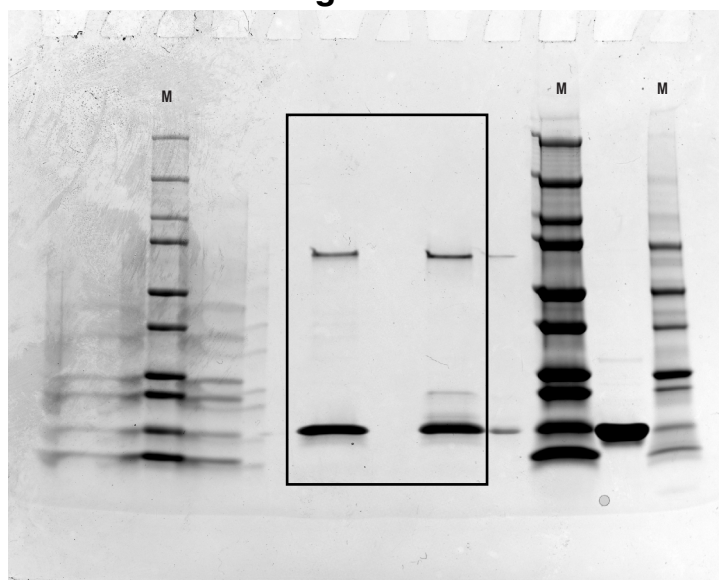

Supplement: SourceData FS2 — is the source file for Fig. S2. [file JCB_202306142_SourceDataFS2.pdf]

**Figure S3A**

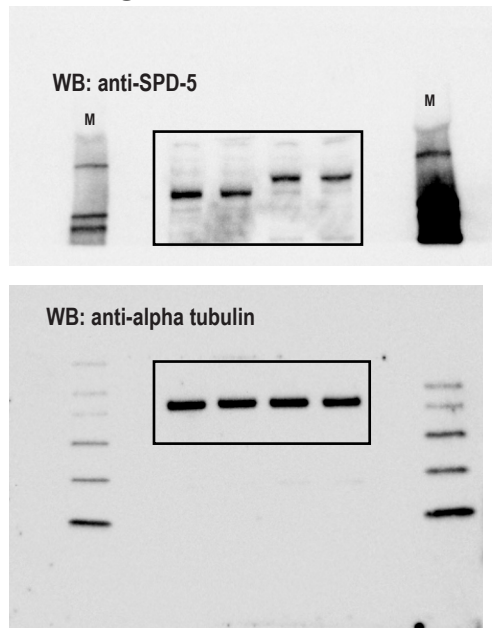

**Figure S3B**

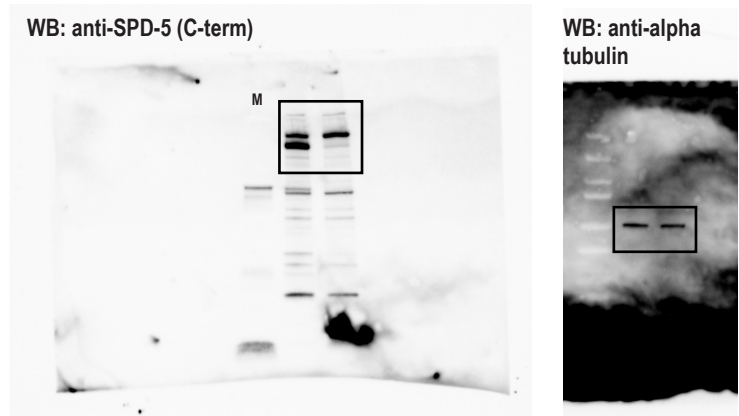

**Figure S3E**

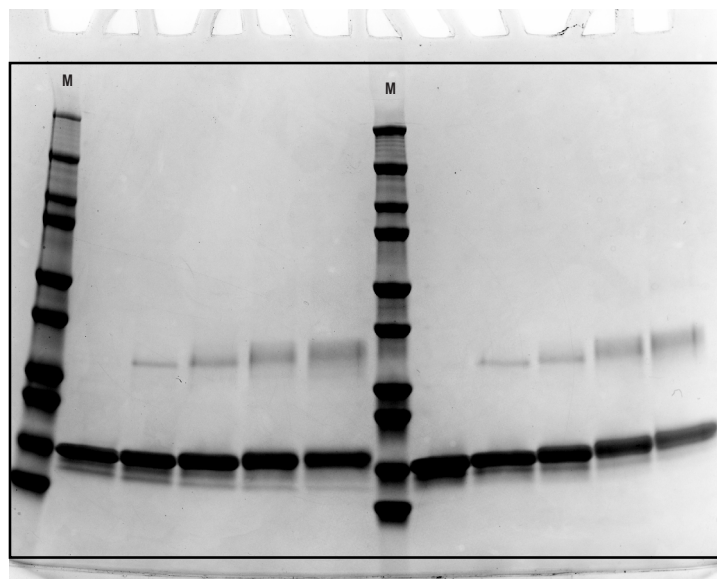

Supplement: SourceData FS3 — is the source file for Fig. S3. [file JCB_202306142_SourceDataFS3.pdf]

**Figure S4B**

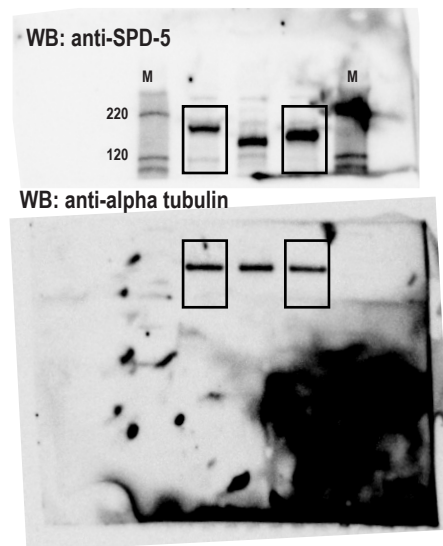

Supplement: SourceData FS4 — is the source file for Fig. S4. [file JCB_202306142_SourceDataFS4.pdf]

**Figure S5B**

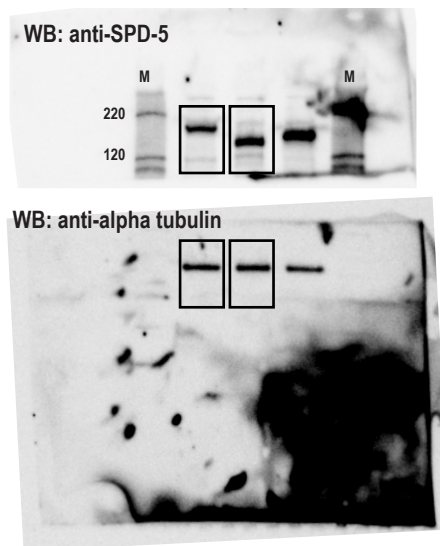

Supplement: SourceData FS5 — is the source file for Fig. S5. [file JCB_202306142_SourceDataFS5.pdf]

**Figure S6B**

WB: anti-GFP

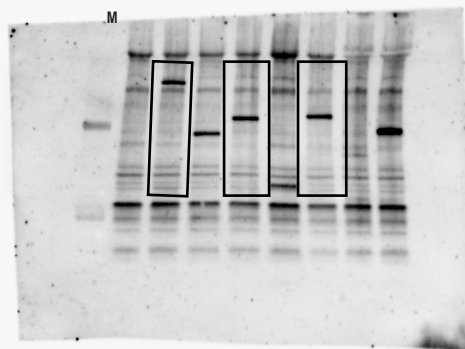

WB: anti-alpha tubulin

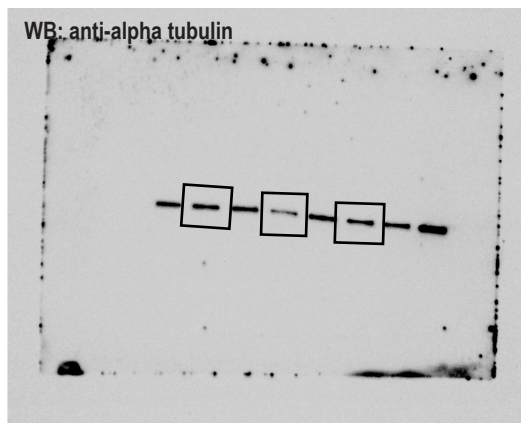

Supplement: SourceData FS6 — is the source file for Fig. S6. [file JCB_202306142_SourceDataFS6.pdf]

**Figure S7A**

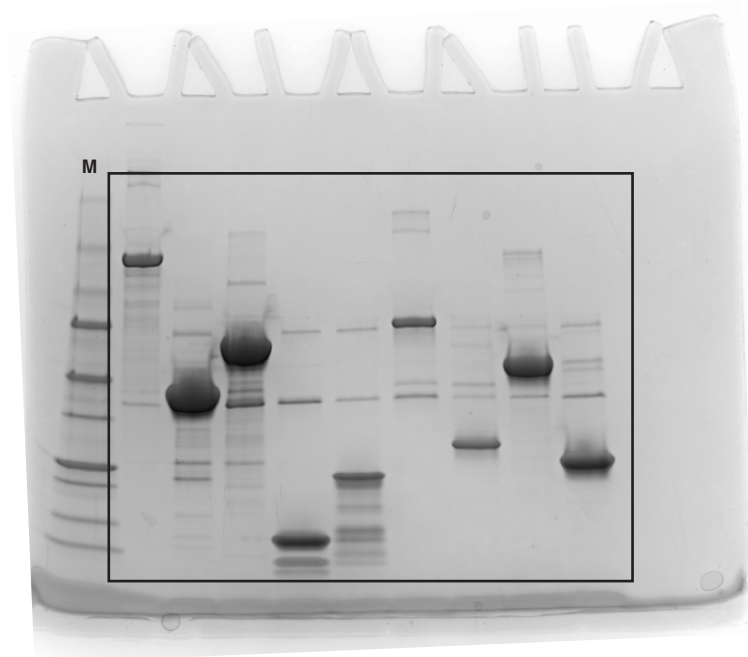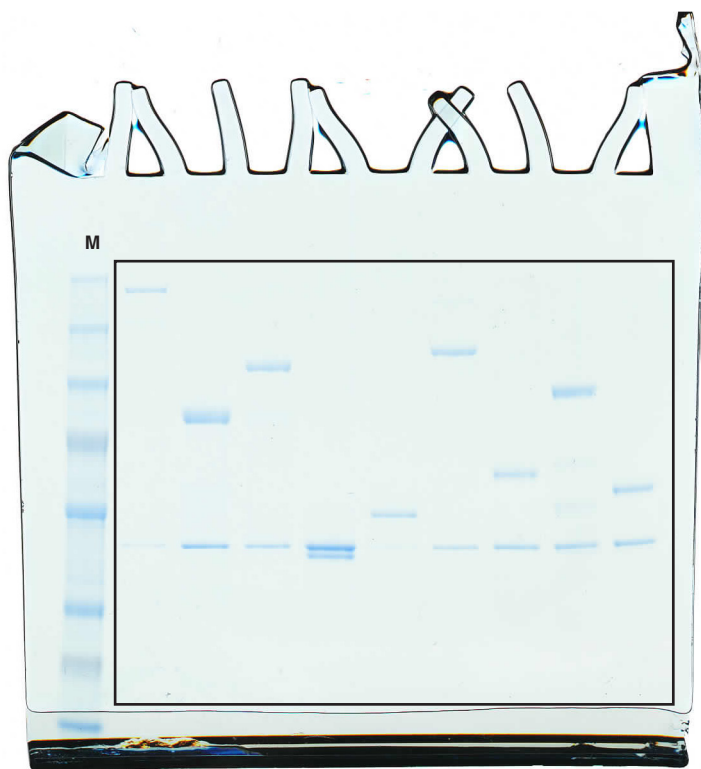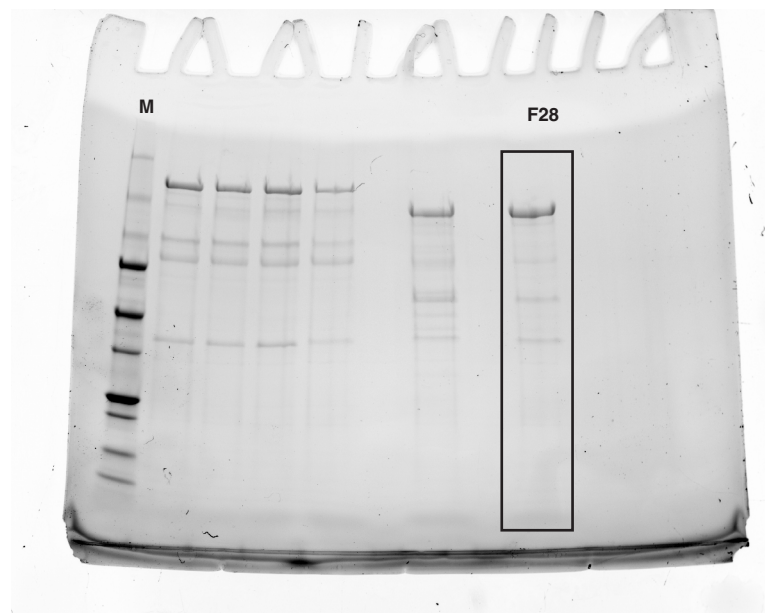

Supplement: SourceData FS7 — is the source file for Fig. S7. [file JCB_202306142_SourceDataFS7.pdf]
